# Supplementary material for: Covalently reactive microparticles imbibe blood to form fortified clots for rapid hemostasis and prevention of rebleeding
Source: Nat Commun. 2025 Apr 18;16:3705. doi: 10.1038/s41467-025-58204-8 (PMC12008190; doi:10.1038/s41467-025-58204-8)
Supplement: Supplementary file 1 — Supplementary Information [file 41467_2025_58204_MOESM1_ESM.pdf]

Supplementary Information for

Covalently reactive microparticles imbibe blood to form fortified clots for rapid hemostasis and prevention of rebleeding

Ting Chen[1], Chaonan Xiao[1], Xianjun Chen[2], Ziyi Yang[3,4], Jingwei Zhao[1,3,4], Bingkun Bao[1], Qingmei Zeng[1], Li Jiang[1], Xinyi Huang[1], Yi Yang[2], Qiuning Lin[1]*, Wei Gong[3,4], Linyong Zhu[1]*

1. School of Biomedical Engineering, Shanghai Jiao Tong University, Shanghai, China
2. Optogenetics & Synthetic Biology Interdisciplinary Research Center, State Key Laboratory of Bioreactor Engineering, East China University of Science and Technology, Shanghai, China.
3. Department of General Surgery, Xinhua Hospital, affiliated to Shanghai Jiao Tong University School of Medicine, Shanghai, China.
4. Shanghai Key Laboratory of Biliary Tract Disease Research, Shanghai, China.

*Corresponding authors. Emails: qiuninglin@sjtu.edu.cn, linyongzhu@sjtu.edu.cn

Contents

1. Supplementary Figures 1-19
2. Supplementary References

Supplementary Figures

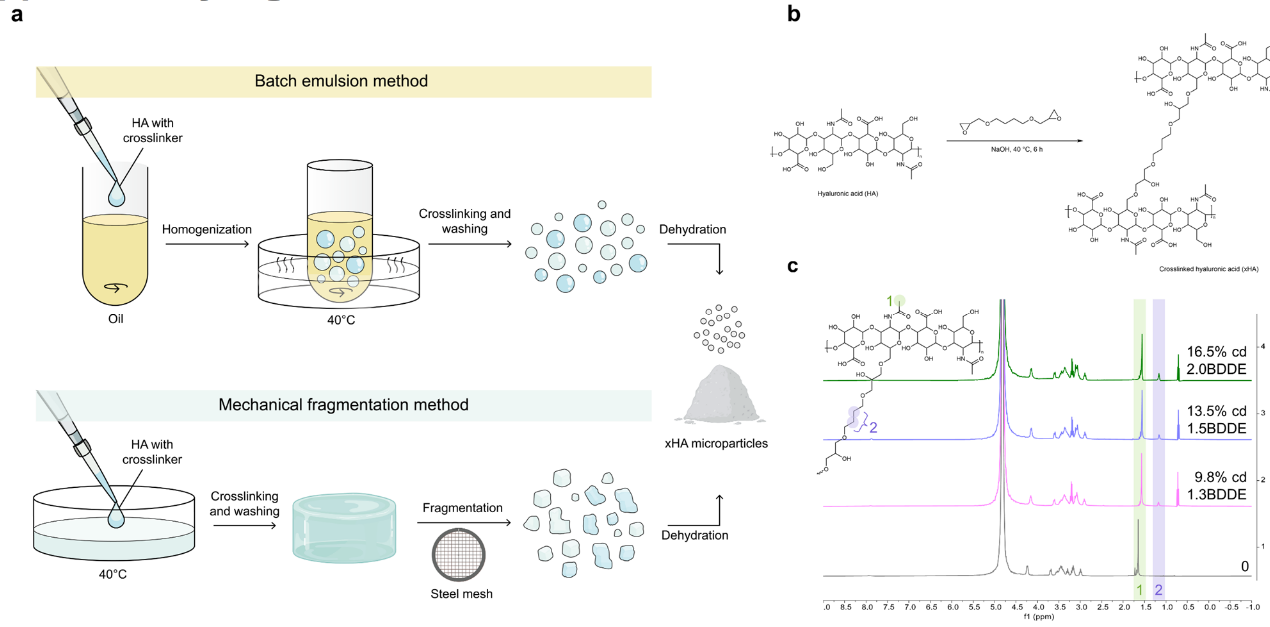

Supplementary Fig.1 | Preparation and characterization of xHA microparticles. a,**b**, Schematic preparation (**a**) and synthetic route (**b**) of xHA microparticles. Some cartoons were created in BioRender. Chen, T. (2025) https://BioRender.com/b67q289. **c**, ^{1}H-Nuclear magnetic resonance (NMR, D_2O, 400 MHz) spectra of xHA microparticles with different feeding ratios of 1,4-Butanediol diglycidyl ether (BDDE). All microparticles were degraded into a solution with sulfuric acid before ^{1}H nuclear magnetic resonance (NMR) analysis. The crosslinking density was defined as the percentage of BDDE per disaccharide. The resonance of ethyl protons (4H) of BDDE appeared at δ ~1.2, and the resonance of methyl protons of HA appeared at δ ~1.6.

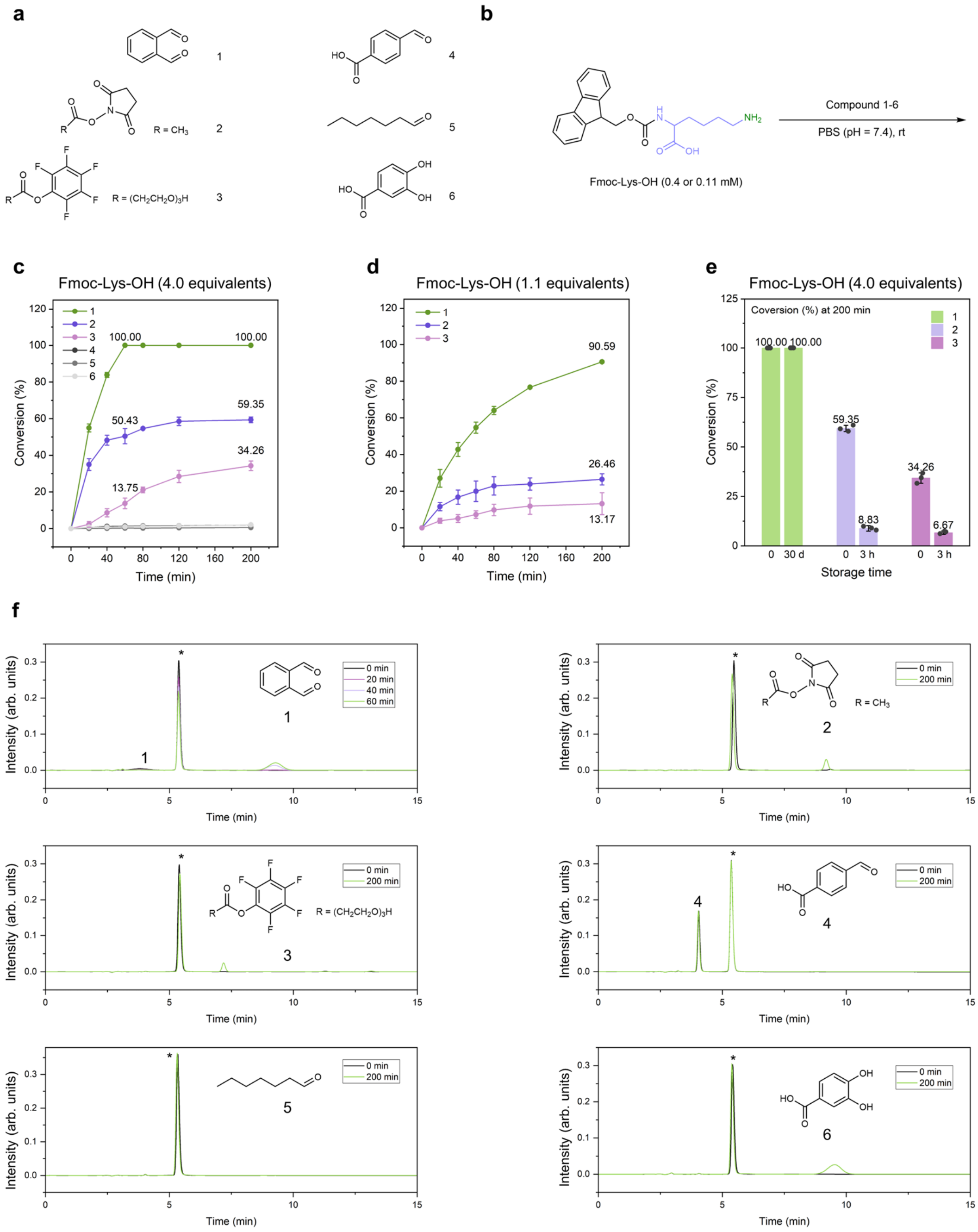

Supplementary Fig.2 | Reactivity and stability evaluation of representative reactive compounds. a, Chemical structures of representative compounds with reactive groups. **b**, Conditions of the reaction between α-*N*-(9-Fluorenylmethyloxycarbonyl)-lysine (Fmoc-Lys-OH) and compound 1-6. **c**,**d**, Conversion of compounds (1.0 equivalent) reacted with Fmoc-Lys-OH (**c**, 4.0 equivalents; **d**, 1.1 equivalents) in phosphate-buffered saline (PBS, pH = 7.4). **e**, Stability evaluation of representative compounds through comparison of conversion (after 200 minutes) before and after storing at aqueous solution (PBS, pH = 7.4) for defined periods of time (30 days for compound 1, and 3 hours for compounds 2 and 3, respectively). **f**, High-performance liquid chromatography (HPLC) spectra for different mixtures in PBS (pH = 7.4) after various incubation times. The initial concentration of Fmoc-Lys-OH (4.0 equivalents) and compounds (1.0 equivalent) in (**e**) and (**f**) was 0.4 mM and 0.1 mM, respectively. The asterisk indicates the unreacted Fmoc-Lys-OH. Data

in (**c**)-(**e**) are presented as means ± s.d. (*n* = 3 independent samples). Source data are provided as a Source Data file.

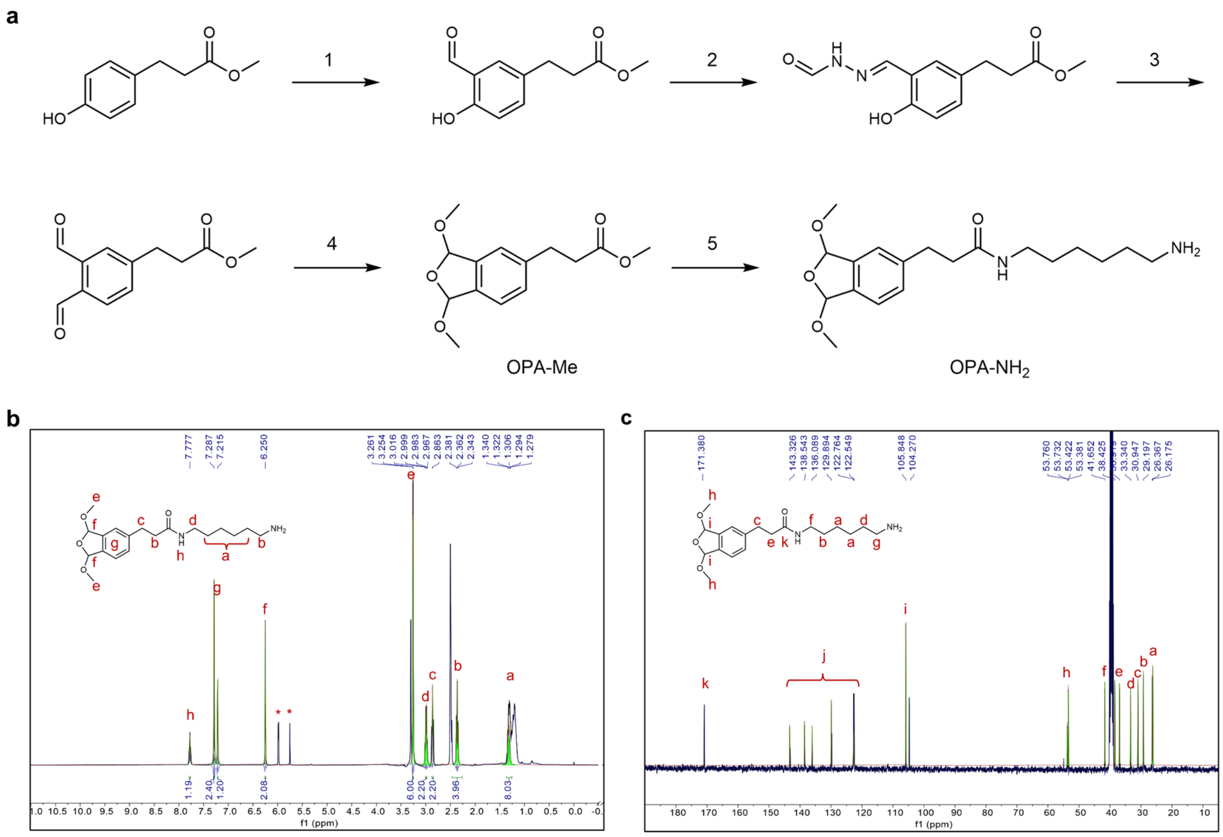

Supplementary Fig.3 | Synthesis and characterization of compound OPA-NH$_2$. a, Synthesis route of compound OPA-NH$_2$. Steps 1 to 4 was performed as the reference[1]. Step 1: acetonitrile, anhydrous magnesium chloride, triethylamine, paraformaldehyde, 80 °C. Step 2: ethanol, formylhydrazine, 80 °C. Step 3: anhydrous tetrahydrofuran (THF), lead tetraacetate, 0 °C. Step 4: THF, lithium tetrafluoroborate, trimethoxymethane, 70 °C. Step 5: *N,N*-dimethylformamide, 1,5,7-triazabicyclo [4.4.0] dec-5-ene, hexamethylenediamine, room temperature. **b**,**c**, ^{1}H-NMR (d_6-DMSO, 400 MHz, **b**) and ^{13}C-NMR (d_6-DMSO, 400 MHz, **c**) spectra of compound OPA-NH$_2$. ^{1}H NMR (400 MHz, DMSO-d_6) δ 7.78 (s, 1H), 7.29 (s, 2H), 7.22 (s, 1H), 6.25 (s, 2H), 3.26 (d, J = 2.6 Hz, 6H), 2.99 (d, J = 6.4 Hz, 2H), 2.86 (s, 2H), 2.36 (t, J = 7.6 Hz, 4H), 1.36 – 1.25 (m, 8H). ^{13}C NMR (101 MHz, DMSO) δ 171.38, 143.33, 138.54, 136.09, 129.89, 122.76, 122.55, 105.85, 104.27, 53.76, 53.73, 53.42, 53.38, 41.65, 38.42, 36.92, 33.34, 30.95, 29.20, 26.37, 26.17.

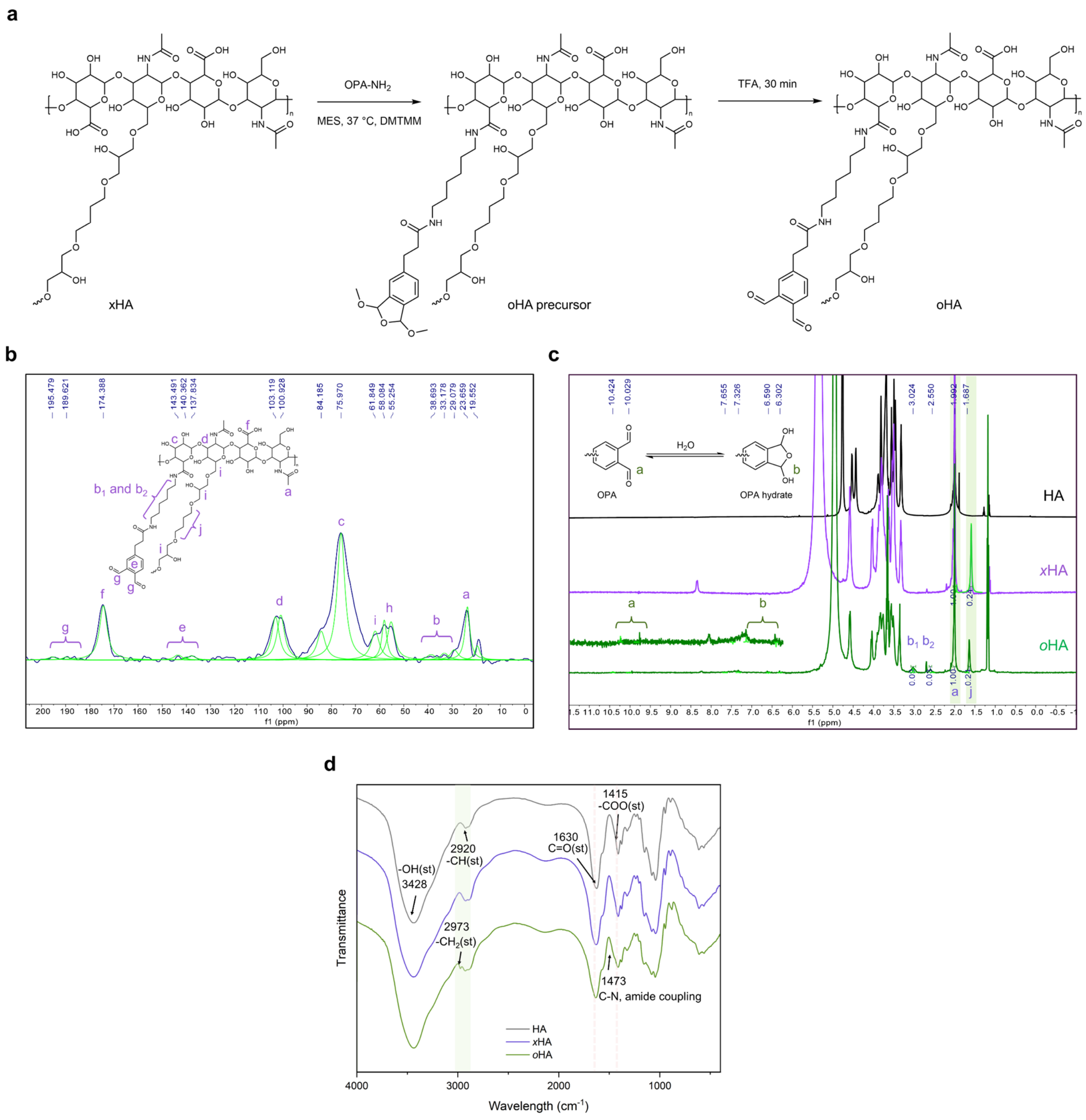

Supplementary Fig.4 | Synthesis and characterization of oHA microparticles. a, Synthesis route of oHA microparticles. **b-d**, Characterization of oHA microparticles. **b**, ^{13}C-NMR (600 MHz) spectrum of oHA microparticles. **c**, ^{1}H-NMR (D_2O, 400 MHz) of HA polymer, xHA, and oHA microparticles. **d**, The Fourier Transform Infrared (FTIR) spectra of HA, xHA, and oHA microparticles. All microparticles were degraded into a solution with sulfuric acid before NMR analysis. The inset shows a vertically stretched view of the region on the left side. The emerging peak at 1.521 ppm was observed in both the xHA and oHA spectra, attributed to the $-CH_2$ of BDDE. The chemical shifts assigned to OPA and its hydrated were observed at 9.86, 10.26 ppm, and 6.14, 6.43 ppm, respectively. In the FTIR spectra, the characteristic peaks of HA, xHA, and oHA include O-H stretching (3428 cm^{-1}), C-H stretching (2920 cm^{-1}), C=O stretching (1630 cm^{-1}), and -COO stretching (1415 cm^{-1}). Compared to HA and xHA, the spectrum of oHA showed two additional absorption

peaks between 2980 and 2880 cm^{-1}, likely corresponding to the introduction of the dialdehyde group[2,3]. The slight decrease in peaks at 1630 cm^{-1} and 1415 cm^{-1}, along with the appearance of a peak found at 1473 cm^{-1}, indicates that OPA groups substitute carboxyl groups and form amide couplings.

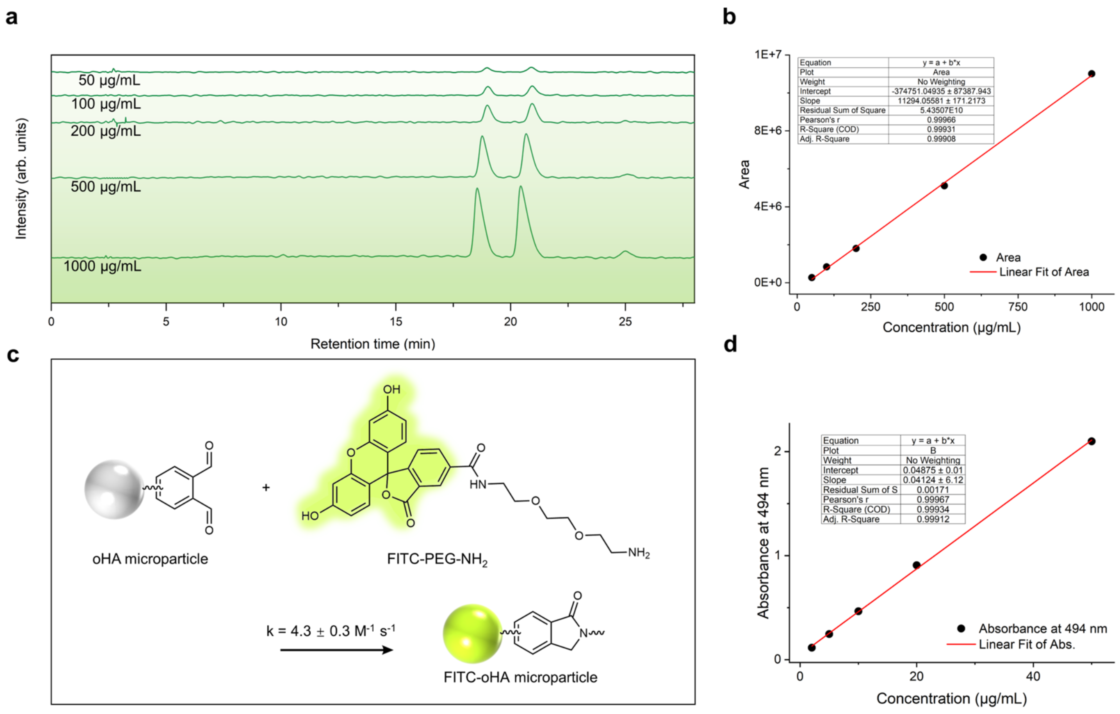

Supplementary Fig.5 | Quantification of OPA substitution degree on oHA microparticles. a-b, Monitoring the modification process of oHA microparticles through HPLC spectra of supernatants. HPLC spectra (**a**) and calibration curve (**b**) of OPA-NH_2. **c**,**d**, Quantifying the OPA substitution degree on oHA microparticles by absorbance differential method. **c**, Schematic illustration of the absorbance differential method. **d**, Calibration curve of FITC-PEG-NH_2 with different concentrations. Source data are provided as a Source Data file.

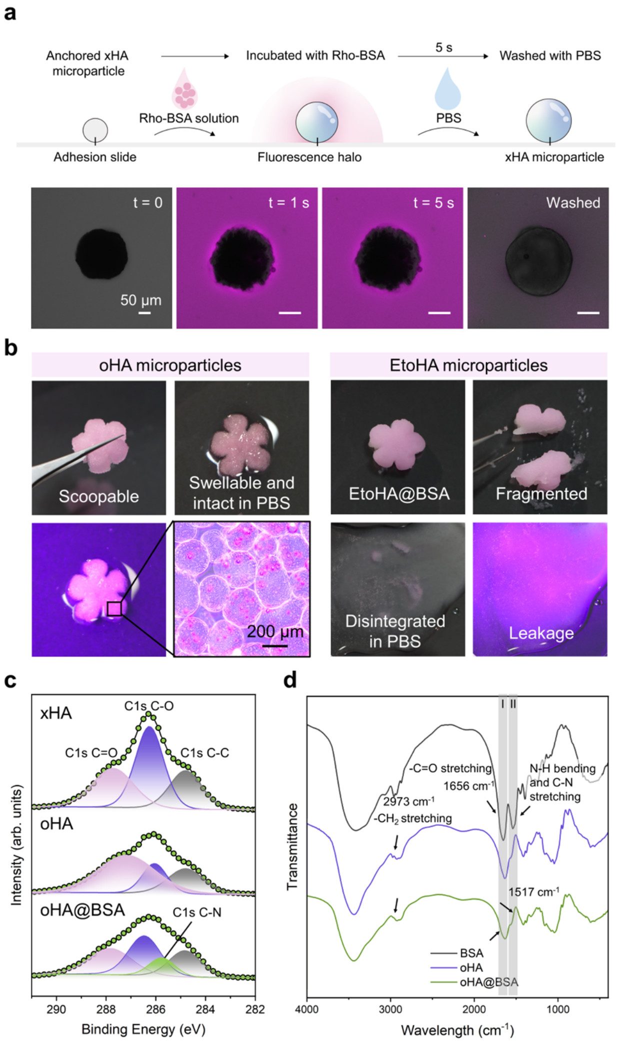

Supplementary Fig.6 | Extra evaluation of imbibition and crosslinking behaviors of oHA microparticles. a, Visualization of the imbibition behavior and no reaction between Rho-BSA (50 mg mL^{-1}, PBS, pH = 7.4) and xHA microparticles. Schematic illustration (up) and CLSM images (below) show the procedure and results, respectively. **b**, Visualization of crosslinking behaviors of oHA microparticles and EtoHA microparticles. **c**,**d**, Characterization of covalent reaction between BSA and oHA microparticles. **c**, Peak-fitting X-ray photoelectron spectroscopy (XPS) spectra in the C1s regions of xHA, oHA, and oHA@BSA microparticles. **d**, The FTIR spectra of BSA, oHA microparticles, and oHA@BSA microparticles.

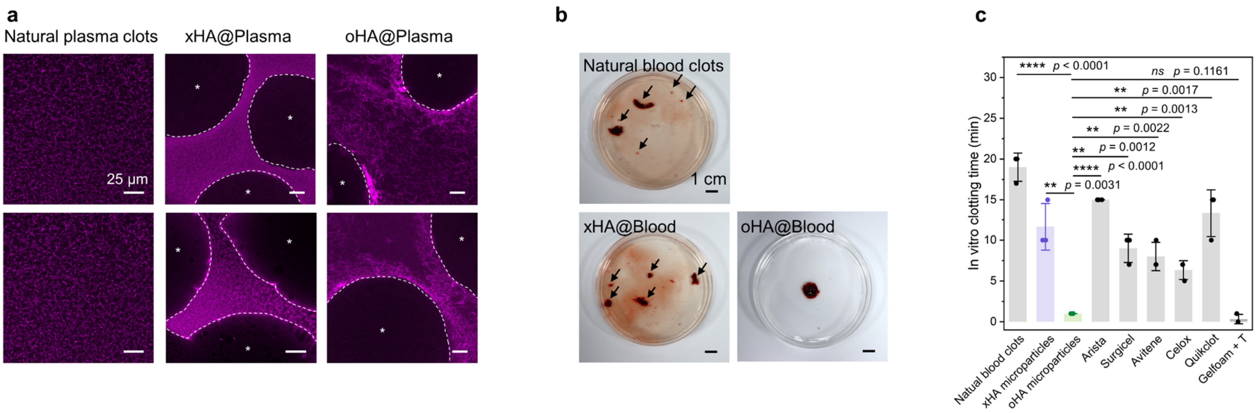

Supplementary Fig.7 | Extra evaluations on acceleration robust clot composite formation and stabilization of oHA microparticles. a, Representative CLSM images of the natural plasma clots, xHA@Plasma, and oHA@Plasma clots. Purple fluorescence corresponds to the rhodamine-labeled plasma proteins. Asterisks indicate the xHA or oHA microparticles; white dashed lines indicate the surface of microparticles. **b**, Representative digital photographs of clots immersed in PBS. The natural blood clot and the xHA@Blood clot composite were disrupted in PBS. The oHA@Blood clot composite was immersed in PBS without disintegration. Black arrows indicate the debris of clots. **c**, The blood clotting time of natural blood clots, and the blood incubated with oHA or xHA microparticles, and commercially available hemostats (i.e., Arista, Surgicel, Avitene, Celox, Quikclot, and Gelfoam +T). 100 μL of calcium chloride ($CaCl_2$, 0.2 M) was added into 1 mL of citrated blood to get the blood. Data are presented as means ± s.d. (n = 3 independent samples). All statistical analyses were performed using an unpaired two-tailed Student t-test. * $p < 0.05$, ** $p < 0.01$, *** $p < 0.001$, **** $p < 0.0001$. Source data are provided as a Source Data file.

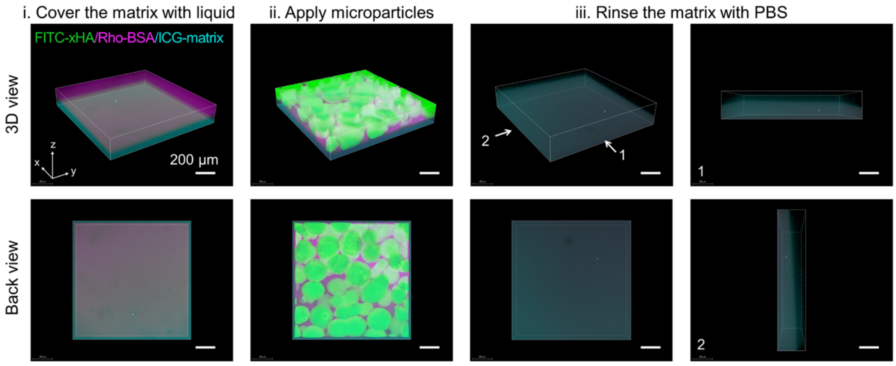

Supplementary Fig.8 | Representative 3D CLSM images show no covalent adhesion between xHA microparticles and Rho-BSA solution (50 mg mL^{-1} in PBS, pH = 7.4)-covered gelatin matrix.

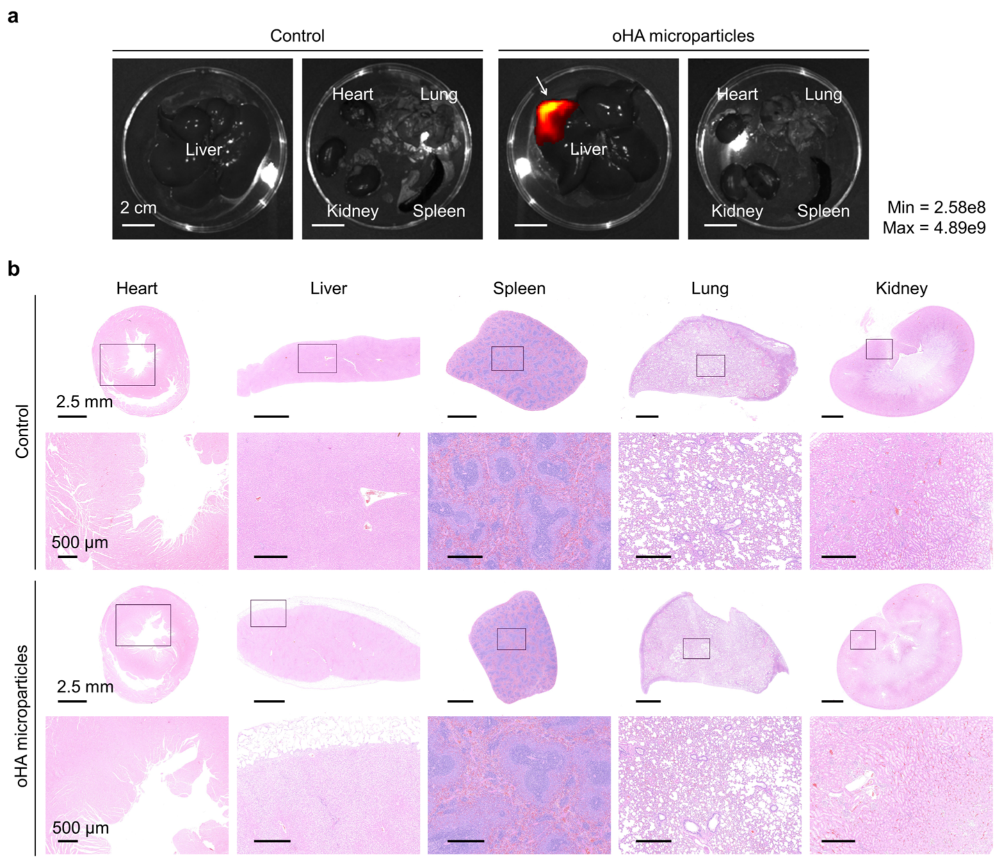

Supplementary Fig.9 | Evaluation of embolic risk after applying oHA microparticles on the rat liver laceration model. Representative (**a**) fluorescence images and (**b**) histological images with H&E staining of ex vivo organs and from a control rat and a rat with oHA microparticles implanted 14 days post-surgery.

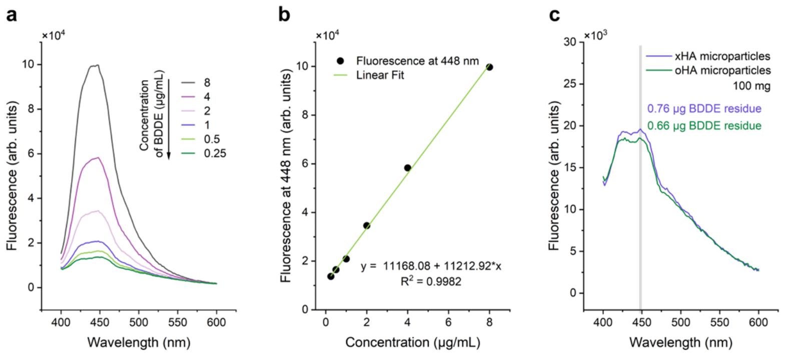

Supplementary Fig.10 | Quantification of residue BDDE crosslinkers. a, Fluorescence emission spectra of the combination with varying concentrations of BDDE. **b**, Calibration curve of BDDE. **c**, Fluorescence emission spectra of enzymatic degradation products from xHA and oHA microparticles (100 mg), characterized by fluorospectrophotometry. Source data are provided as a Source Data file.

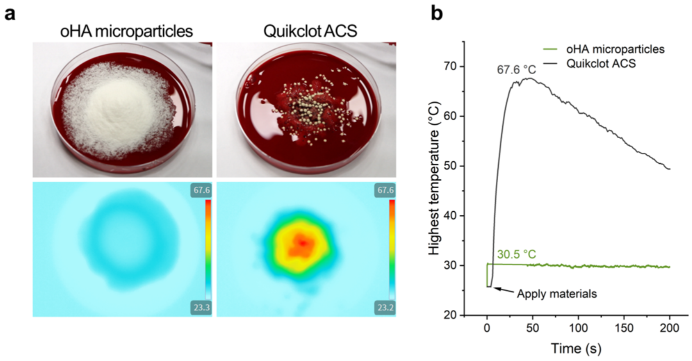

Supplementary Fig.11 | Evaluation of an exothermic reaction. a, Digital photos (top) and infrared thermal images of oHA microparticles and Quikclot ACS (bottom) contacting blood. A total of 10 mL of citrated whole blood was added into a clear dish (diameter: 80 mm), and 2 g of oHA microparticles and Quiklclot (Advanced clotting sponge) were added to the dish. Dishes were placed at room temperature, and the highest temperature within the dish was recorded using an infrared thermometer (Fortric, Fixed-mount 600 Series). **b**, Temperature curves showing the highest recorded temperature in the dish over time. Source data are provided as a Source Data file.

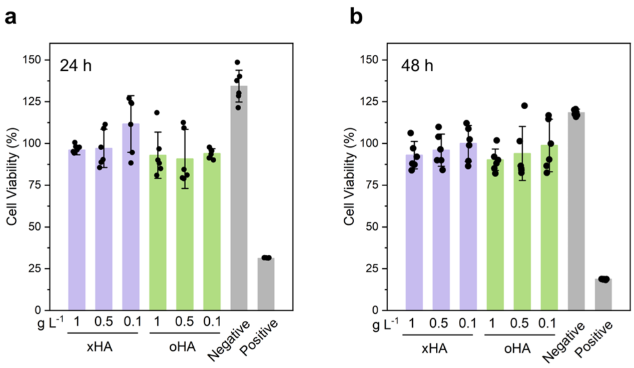

Supplementary Fig.12 | In vitro cell viability of mouse fibroblast (L929) cells assessed by CCK8 assays after incubation with different DMEM for (a) 24 hours and (b) 48 hours. Data are presented as means ± s.d. (*n* = 6 independent samples). Source data are provided as a Source Data file.

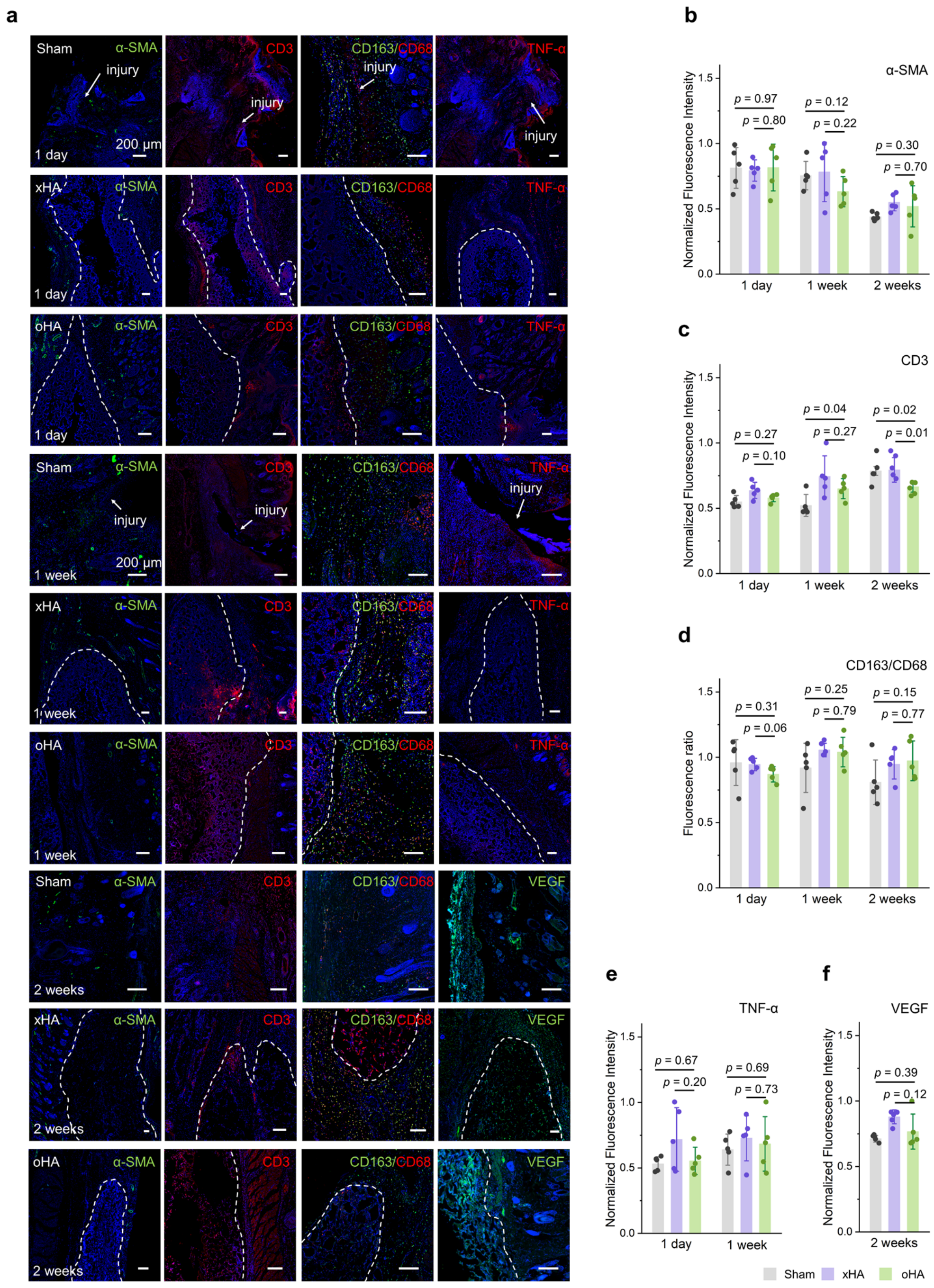

Supplementary Fig.13 | Extra in vivo biocompatibility evaluation via Immunofluorescence staining of the subcutaneous tissue. a, Representative immunofluorescence images 1 day, 1 week, and 2 weeks after implantation. Cell nuclei are stained with 4',6-diamidino-2-phenylindole (DAPI, blue). Green fluorescence corresponds to the expression of fibroblasts (α-SMA) and M2 macrophages (CD163). Red fluorescence corresponds to the expression of T cells (CD3), TNF-α, and macrophages (CD68). **b**,**c**, Normalized fluorescence intensity of α-SMA (**b**) and CD3 (**c**) 1 day, 1 week, and 2 weeks after implantation. **d**, Normalized fluorescence ratio of CD163/CD68 1 day ,1 week, and 2 weeks after implantation. **e**, Normalized fluorescence intensity of TNF-α 1 day and 1 week after implantation. **f**, Normalized fluorescence intensity of VEGF 2 weeks after implantation. Data in (**b**)-(**f**) are presented as means ± s.d. (*n* = 5 independent samples). All statistical

analyses were performed using an unpaired two-tailed Student *t*-test. * $p < 0.05$, ** $p < 0.01$, *** $p < 0.001$, **** $p < 0.0001$. Source data are provided as a Source Data file.

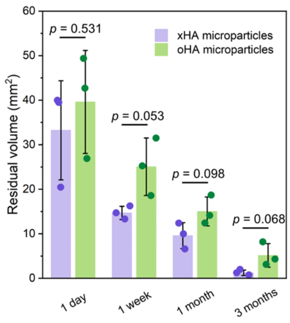

Supplementary Fig.14 | Residue volume of xHA and oHA microparticles in the rat subcutaneous implantation model. Data are presented as means ± s.d. ($n = 3$ independent samples), and all statistical analyses were performed using an unpaired two-tailed Student *t*-test. * $p < 0.05$, ** $p < 0.01$, *** $p < 0.001$, **** $p < 0.0001$. Source data are provided as a Source Data file.

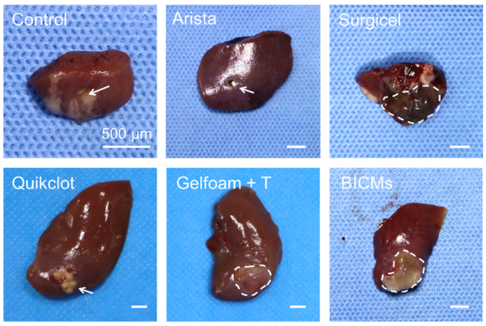

Supplementary Fig.15 | Representative digital photos of the injured livers one week after injury.

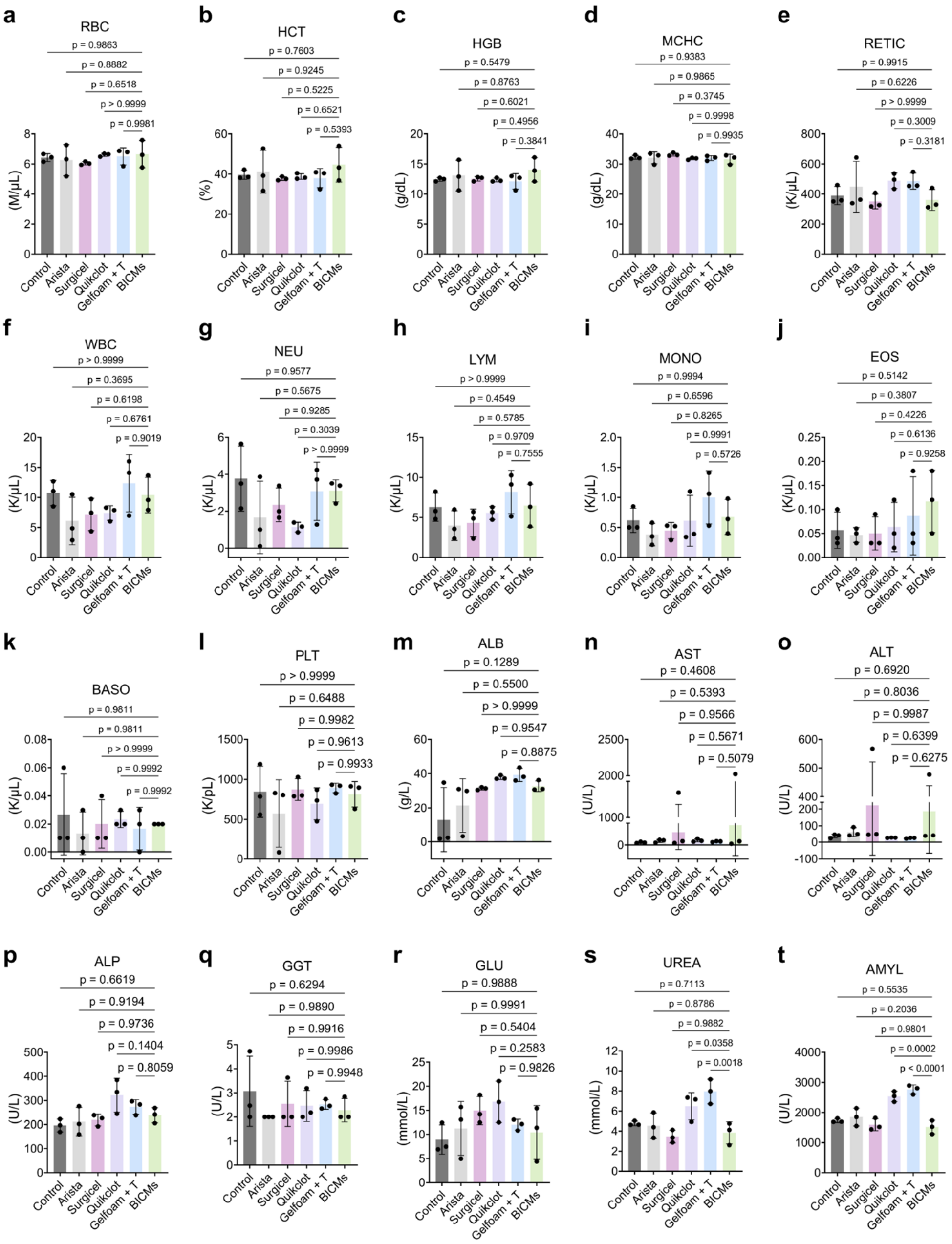

Supplementary Fig.16 | Blood analysis of the rats 1 week after hemostasis. a-l, Complete blood count (CBC) of the rats 1 week after hemostasis for red blood cell (RBC, **a**), hematocrit (HCT, **b**), hemoglobin (HGB, **c**), mean corpuscular hemoglobin concentration (MCHC, **d**), reticulocyte (RETIC, **e**), white blood cell (WBC, **f**), neutrophil (NEU, **g**), lymphocyte (LYM, **h**), monocyte (MONO, **i**), eosinophil (EOS, **j**), basophil (BASO, **k**), and platelet (PLT, **l**). **m-t**, Blood chemistry of the rats 1 week after hemostasis for albumin (ALB, **m**), aspartate transaminase (AST, **n**), alanine aminotransferase (ALT, **o**), alkaline phosphatase (ALP, **p**), gamma-glutamyl transferase (GGT, **q**), glucose (GLU, **r**), urea (**s**), and amylase (AMYL, **t**). Data are presented as means ± s.d. (*n* = 3 independent samples). All statistical analyses were performed using a one-way ANOVA test. * *p* < 0.05, ** *p* < 0.01, *** *p* < 0.001, **** *p* < 0.0001. Source data are provided as a Source Data file.

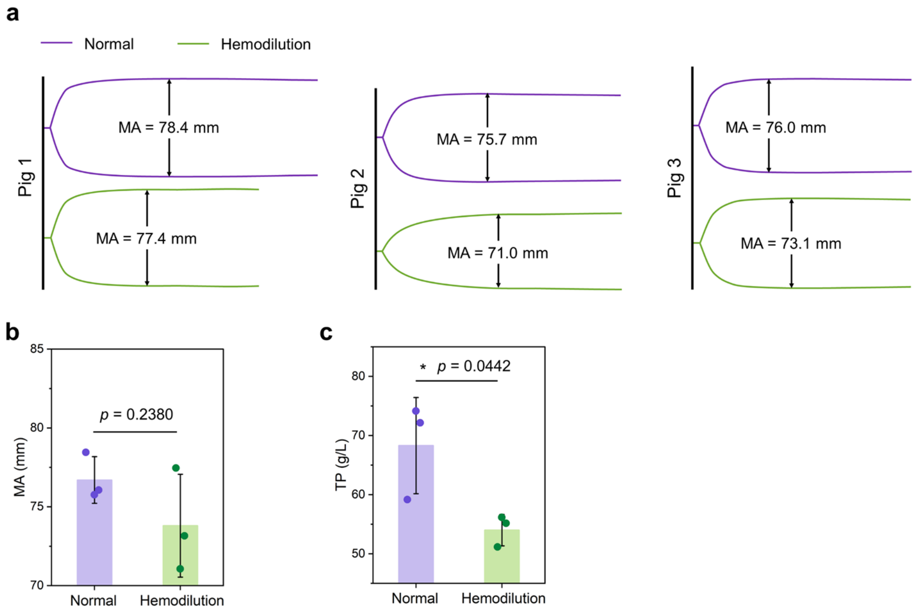

Supplementary Fig.17 | Validation of the hemodilution-induced coagulopathic pig model. a, Thromboelastograms (TEGs) of pigs before (normal) and after hemodilution. **b**,**c**, Maximum amplitude (MA) values (**b**) and Total protein (TP) concentration (**c**) of pigs before (normal) and after hemodilution. Data in (**b**) and (**c**) are presented as means ± s.d. (*n* = 3 independent samples). All statistical analyses were performed using an unpaired two-tailed Student *t*-test. * $p < 0.05$, ** $p < 0.01$, *** $p < 0.001$, **** $p < 0.0001$. Source data are provided as a Source Data file.

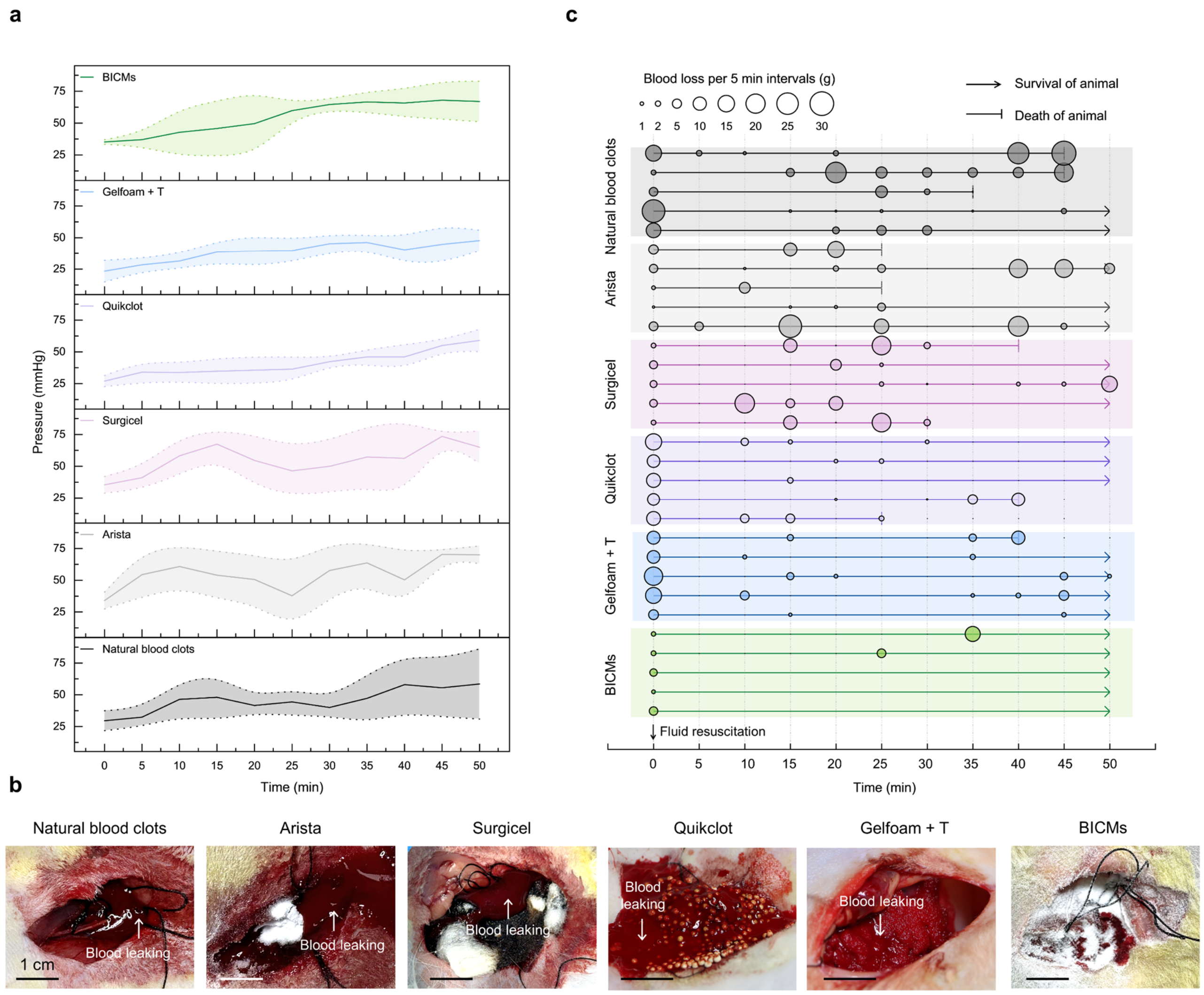

Supplementary Fig.18 | Extra evaluations of *in vivo* hemostatic performance in rabbit femoral artery injury model. a, Mean arterial blood pressure in the 50 minutes of fluid resuscitation. **b**, Representative photos of failed and successful hemostasis treated with natural blood clots, Arista, Surgicel, Quikclot, Gelfoam + T, and BICMs. **c**, The rebleeding profiles of treated rabbits, where each line represents an individual animal. Data in (**a**) are presented as means ± s.d. (*n* = 5 independent samples). Source data are provided as a Source Data file.

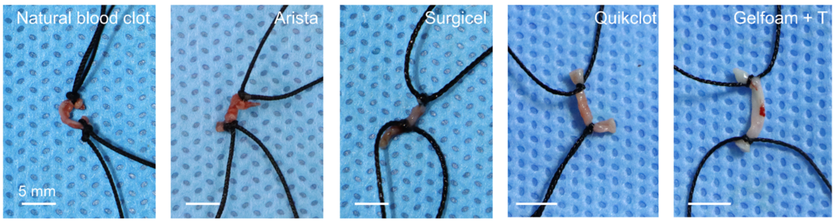

* Sutures were ligated after euthanasia.

Supplementary Fig.19 | Representative digital photos of the collected femoral arteries in the natural blood clot, Arista, Surgicel, Quikclot, and Gelfoam + T group.

Supplementary References

1 Tung, C. L., Wong, C. T. T., Fung, E. Y. M. & Li, X. Traceless and Chemoselective Amine Bioconjugation via Phthalimidine Formation in Native Protein Modification. *Org. Lett.* **18**, 2600-2603 (2016). https://doi.org:10.1021/acs.orglett.6b00983

2 Zhang, R., Xue, M., Yang, J. & Tan, T. A novel injectable and in situ crosslinked hydrogel based on hyaluronic acid and α,β-polyaspartylhydrazide. *J. Appl. Polym. Sci.* **125**, 1116-1126 (2012). https://doi.org:https://doi.org/10.1002/app.34828

3 Muhammad, M., Willems, C., Rodríguez-Fernández, J., Gallego-Ferrer, G. & Groth, T. Synthesis and Characterization of Oxidized Polysaccharides for In Situ Forming Hydrogels. *Biomolecules* **10**, 1185 (2020).
