# Supplementary material for: Covalently reactive microparticles imbibe blood to form fortified clots for rapid hemostasis and prevention of rebleeding
Source: Nat Commun. 2025 Apr 18;16:3705. doi: 10.1038/s41467-025-58204-8 (PMC12008190; doi:10.1038/s41467-025-58204-8)
Supplement: Supplementary file 2 — Description of Additional Supplementary Files [file 41467_2025_58204_MOESM2_ESM.pdf]

Description of Additional Supplementary Files

File Name: Supplementary Movie 1

Description: The trajectory of oHA microparticles from above the liquid to the deep and narrow bottom. The liquid was a transparent protein solution (BSA, indicated by a yellow dye, 50 mg mL^{-1}).

File Name: Supplementary Movie 2

Description: Adhesion performance of natural clots, Arista, Surgicel, Avitene, Celox, Quikclot, Gelfoam + T, xHA, EtoHA and oHA microparticles under gradually raising hydrophilic pressure. The pressure was varied and monitored by a water pressure gauge with a release valve. The video was played at quadruple speed.

File Name: Supplementary Movie 3

Description: Adhesion performance of an oHA@Blood clot on a fresh and punched porcine aorta. The circulating BSA solution (50 mg mL^{-1}, PBS, pH = 7.4) was supplemented with rhodamine as an indicator. The video was played at quadruple speed.

File Name: Supplementary Movie 4

Description: A rabbit femoral artery injury hemostasis by BICMs during fluid resuscitation. The video was initially played at double speed, and then at quadruple speed.

File Name: Supplementary Movie 5

Description: Blood leakage appeared under the natural clot, and Arista, Surgicel, Quikclot, or Gelfoam + T participated-clots in the rabbit model of femoral artery hemorrhage and fluid resuscitation. The video was played at quadruple speed.
